# Predicting COPD 1-year mortality using prognostic predictors routinely measured in primary care

**DOI:** 10.1186/s12916-019-1310-0

**Published:** 2019-04-05

**Authors:** C. I. Bloom, F. Ricciardi, L. Smeeth, P. Stone, J. K. Quint

**Affiliations:** 1 National Heart Lung Institute, Imperial College London, Emmanuel Kaye Building, 1b Manresa Road, London, SW3 6LR UK; 2 Department of Statistical Science, University College London, London, UK; 3 Epidemiology and Population Health, London School of Hygiene and Tropical Medicine, LSHTM, Keppel Street, London, WC1E 7HT UK; 4 Marie Curie Palliative Care Research Department, University College London, London, UK; 5 Department of Respiratory Epidemiology, Occupational Medicine and Public Health, NHLI, Imperial College London, London, UK

**Keywords:** COPD, Prediction, Risk score, Mortality, Palliative care

## Abstract

**Background:**

Chronic obstructive pulmonary disease (COPD) is a major cause of mortality. Patients with advanced disease often have a poor quality of life, such that guidelines recommend providing palliative care in their last year of life. Uptake and use of palliative care in advanced COPD is low; difficulty in predicting 1-year mortality is thought to be a major contributing factor.

**Methods:**

We identified two primary care COPD cohorts using UK electronic healthcare records (Clinical Practice Research Datalink). The first cohort was randomised equally into training and test sets. An external dataset was drawn from a second cohort. A risk model to predict mortality within 12 months was derived from the training set using backwards elimination Cox regression. The model was given the acronym BARC based on putative prognostic factors including body mass index and blood results (B), age (A), respiratory variables (airflow obstruction, exacerbations, smoking) (R) and comorbidities (C). The BARC index predictive performance was validated in the test set and external dataset by assessing calibration and discrimination. The observed and expected probabilities of death were assessed for increasing quartiles of mortality risk (very low risk, low risk, moderate risk, high risk). The BARC index was compared to the established index scores body mass index, obstructive, dyspnoea and exacerbations (BODEx), dyspnoea, obstruction, smoking and exacerbations (DOSE) and age, dyspnoea and obstruction (ADO).

**Results:**

Fifty-four thousand nine hundred ninety patients were eligible from the first cohort and 4931 from the second cohort. Eighteen variables were included in the BARC, including age, airflow obstruction, body mass index, smoking, exacerbations and comorbidities. The risk model had acceptable predictive performance (test set: C-index = 0.79, 95% CI 0.78–0.81, D-statistic = 1.87, 95% CI 1.77–1.96, calibration slope = 0.95, 95% CI 0.9–0.99; external dataset: C-index = 0.67, 95% CI 0.65–0.7, D-statistic = 0.98, 95% CI 0.8–1.2, calibration slope = 0.54, 95% CI 0.45–0.64) and acceptable accuracy predicting the probability of death (probability of death in 1 year, *n* high-risk group, test set: expected = 0.31, observed = 0.30; external dataset: expected = 0.22, observed = 0.27). The BARC compared favourably to existing index scores that can also be applied without specialist respiratory variables (area under the curve: BARC = 0.78, 95% CI 0.76–0.79; BODEx = 0.48, 95% CI 0.45–0.51; DOSE = 0.60, 95% CI 0.57–0.61; ADO = 0.68, 95% CI 0.66–0.69, external dataset: BARC = 0.70, 95% CI 0.67–0.72; BODEx = 0.41, 95% CI 0.38–0.45; DOSE = 0.52, 95% CI 0.49–0.55; ADO = 0.57, 95% CI 0.54–0.60).

**Conclusion:**

The BARC index performed better than existing tools in predicting 1-year mortality. Critically, the risk score only requires routinely collected non-specialist information which, therefore, could help identify patients seen in primary care that may benefit from palliative care.

**Electronic supplementary material:**

The online version of this article (10.1186/s12916-019-1310-0) contains supplementary material, which is available to authorized users.

## Introduction

Chronic obstructive pulmonary disease (COPD) is associated with significant mortality and morbidity and is one of the most prevalent chronic diseases globally; in the UK, it is the fifth highest cause of death [1, 2]. As COPD progresses, patients experience significant decreases in functional capacity, quality of life, social ability and psychological well-being, impairments that are analogous to those from lung cancer. There is growing evidence and increasing expert opinion that palliative care should have a prominent role in patients with end-stage COPD [3, 4]. UK clinical guidelines (National Health Service, National Institute for Health and Care Excellence, National Council for Palliative Care) all recommend starting palliative care in the year before people die, with the goal of both improving their quality of life and addressing end-of-life planning [3, 5]. The healthcare workers best placed to enable this are often those in primary care. However, we have previously shown in the UK that only 1 in 5 COPD patients within the last year of life are provided palliative care, and a recent Canadian study of COPD patients with advanced disease found a similarly low proportion [6, 7]. One major barrier to provision is the challenge of predicting patient survival, due to the irregular disease trajectory of COPD, which is usually one of slow decline, punctuated by sudden unpredictable exacerbations that often end in death [4, 8–10]. This is in contrast to lung cancer, where there is often a reasonable level of physical function until a short period of relatively predictable decline. This may partly explain why COPD patients are much less likely to receive palliative care than patients with lung cancer [7].

Many derived prognostic indices can help long-term mortality prediction in COPD, but the ability to predict death at 12 months is currently limited, thought in part to be because the original derivation of some of the scores was to predict mortality over several years, as well as the lack of inclusion of important prognostic factors, such as comorbidities [4, 8, 11]. Furthermore, these risk scores have been derived using subgroups of patients, in particular patients from secondary care, where more specialised test results are available. Hence, these indices often cannot be applied to the general COPD population, for example, the BODE index is the most commonly used yet requires knowledge of a patient’s exercise capacity, measured by their 6-min walk test, which is not routinely carried out in a primary care setting. This limitation prevents those that most commonly attend to COPD patients, healthcare workers within primary care, from identifying COPD patients that would benefit from palliative care. Lastly, the simplicity of the most commonly used predictive indexes may impede their predictive ability, such that addition of clinical variables increased their performance [11]. This seems especially relevant when adding comorbidities as putative prognostic predictors; comorbidities such as cardiovascular disease, cerebrovascular disease and lung cancer are both associated with an increased mortality and are highly prevalent in COPD patients. Moreover, there is evidence to suggest COPD patients are more likely to die from their comorbidities than the disease itself [12].

The aim of this study was to devise a prognostic tool, based on routinely collected variables within primary care, which could provide a 12-month mortality prognosis for general COPD patients. To carry this out, we used the UK’s largest longitudinal database of electronic healthcare records and incorporated in our analysis all recorded putative predictive risk factors; these risk factors were based on previous published indices and risk scores.

## Methods

### Data sources

Data from the Clinical Practice Research Datalink (CPRD) was used to derive the prognostic risk model. CPRD currently covers more than 11 million patients, who represent the population, including with respect to gender and age, containing primary care clinical, prescription and test data [13]. To obtain data on exacerbations, socioeconomic status and mortality, linkage respectively to Hospital Episode Statistics (HES), Index of Multiple Deprivation (IMD) and Office of National Statistics (ONS) data was obtained; just over 60% of CPRD practices have patient-level linkage to HES-IMD-ONS.

### Study populations

All patients had a COPD diagnosis as determined using a previously validated algorithm [14]. Patients’ data were eligible for inclusion after the latest of their COPD diagnosis date, the date the GP practice began recording research quality data, their continuous CPRD registration date, or cohort start date. Patients’ data were censored at the earliest of their date of death, end of study (26 June 2015), the GP practice last collection date or the date of transfer out of a CPRD-linked practice. Two study populations were drawn. The first had a cohort start date of 1 January 2010, and an arbitrary index date (time from which the 1-year mortality prognosis model could be applied) set as the first annual COPD review that occurred 12 months after eligibility. This cohort was used to derive the model and internally validate the model.

A second population was drawn that did not have a recorded annual review date and had data drawn from an earlier time period. The second cohort start date was 1 January 2004, and index date was arbitrarily set as the first day after 12 months of eligible data had occurred. Patients were excluded if they had a recorded annual review date between 1 January 2004 and 26 June 2015, and if they had missing values required for the model.

### Outcome and prognostic predictors

Death was defined as mortality from any cause. The following prognostic predictors were chosen, based on published indices and risk scores, using appropriate Read codes (codes are available upon request): history of smoking (current or ex-smoker), MRC dyspnoea score, bereavement, myocardial infarction, asthma, osteoporosis, diabetes, hypertension, dementia, lung cancer, heart failure, stroke, anxiety, depression, atrial fibrillation, pulmonary embolism, coronary artery disease, gastric/duodenal ulcer disease, breast cancer, pancreatic cancer, pulmonary fibrosis, stroke, long-term oxygen therapy, influenza and pneumococcal vaccinations (this can be given every 5 years; if records did not extend beyond 5 years and did not show vaccination, this was recorded as missing) [8]. The COTE score (based on the presence of multiple comorbidities, including lung fibrosis, pancreatic cancer and diabetes with neuropathy) was also calculated [15]. Lung fibrosis was defined as any interstitial lung disease (ILD), e.g. sarcoidosis, idiopathic pulmonary fibrosis, rheumatoid arthritis-associated ILD. Prescription data was used to identify patients that had ever used an inhaled corticosteroid (ICS), long-acting beta agonist (LABA), or long-acting muscarinic antagonist (LAMA). Test results were used to identify the following variables, FEV_1_, GOLD staging (FEV_1_ and FVC), C-reactive protein (CRP), albumin (low = < 35 g/L), haemoglobin, fibrinogen, platelets (low = < 150 × 10^9^/L, high = > 400 × 10^9^/L) and creatinine; creatinine above 120 μmol/L for males, or 110 μmol/L for females, was used to define chronic kidney disease (CKD). BMI was measured as kg/m^2^ (underweight < 19, normal = 19–25, overweight = 25–30, obese ≥ 30). Exacerbations, treated within primary (labelled as moderate) or secondary care (labelled as severe), were identified using a validated algorithm [16, 17]. Severe exacerbations were categorised as none, 1–2 hospitalisations annually and ≥ 3 hospitalisations annually. The rules for variable inclusion are defined in Additional file 1: Table S1.

### Multivariable prognostic scores

Only three of the nine multivariable scores, which have previously been used to address mortality in unselected COPD patients at 1 year, were able to be derived from routinely collected primary care data. These were ADO (age, dyspnoea and airflow obstruction), BODEx (BMI, airflow obstruction, dyspnoea and exacerbations) and DOSE (dyspnoea, airflow obstruction, smoking status and exacerbations). The scores were derived as per original publication, using variables as defined above (MRC dyspnoea score, FEV_1_, smoking status, exacerbations, BMI) [18–20].

### Modelling the putative prognostic predictors

The dataset was randomly divided equally into two datasets: a training set, used to derive the model, and a test set, used to internally validate the risk model.

Variables exceeding 50% missing were excluded from the model. An imputation model was defined for each variable with ≤ 50% missing data. Data were assumed to be missing at random, and values for the missing predictors were imputed using multiple imputation techniques based on chained equations [21]. A total of 10 imputed datasets were generated.

To derive the risk model, Cox regression models were fitted using the data from the training set with all predictors (with the exclusion of the COTE scores). Backwards elimination with a stack approach [21] was used, using a 5% significance level for variable selection and weights equal to 1/10 for each one of the imputed training datasets. The coefficient estimates for the final model were combined from the imputed datasets using Rubin’s rules [22]. Proportional hazard assumptions were tested for the final model.

The probability of mortality at 1 year for a patient can be calculated using the following equation, derived from the Cox proportional hazards model:

$$ P\left(\mathrm{death}\ \mathrm{at}\ 1\ \mathrm{year}\right)=1-{\left({S}_0(t)\right)}^{\exp \left(\mathrm{prognostic}\ \mathrm{index}\right)}, $$

where *S*_0_(*t*) is the baseline survival probability at time *t* (i.e. at 1 year in this study). The prognostic index, i.e. the linear predictor of the Cox model, is the quantity we used as our proposed index. The index was given the acronym BARC based on putative prognostic factors including body mass index and blood results (B), age (A), respiratory variables (airflow obstruction, exacerbations, smoking) (R) and comorbidities (C).

### Validation of the risk model

To validate the predictive ability of the risk model at 12 months, we relied on the calculation of the BARC index in the test set using the coefficients obtained in the development phase. The model was validated internally in the test dataset and in the external dataset (drawn from the second COPD cohort). Measures assessing calibration (calibration slope) and discrimination (Harrel’s C-index and D-statistic) were calculated [23–25]. Calibration slope assesses the agreement between predicted and observed risks. A calibration slope of 1 suggests perfect calibration, while a value diverging from 1 is indicative of poorer agreement. A value of 0.5 for C-index indicates no discrimination, and 1 indicates perfect discrimination. A model with no discriminatory ability will produce D value equal to 0, and better separation is achieved with higher values. The performance measures were estimated in each imputed validation test dataset, overall measures were calculated by combining the estimates using Rubin’s rules, and in the external dataset.

Graphical illustration of calibration is given by comparing observed (Kaplan–Meier) and predicted survival probabilities in several prognostic groups. Groups were derived by placing cut points on the BARC based on meaningful quantiles [26, 27]. We categorised BARC index’s at the 1st quartile, median and 3rd quartile of the time of death, i.e. not counting censored observations, to create four risk groups.

### Comparing observed and predicted mortality probability

The observed mortality probability was calculated by the proportion of deceased patients in the sample within a year. The same four groups used to graphically calibrate the model were used to classify subjects in very low, low, moderate and high risk [28]. Mortality could then be compared for patients in each risk group between that observed and the predicted mortality using the BARC index.

### Comparing the risk model with established multivariable prognostic scores

To compare the predictive capability of the BARC index with that of ADO, BODEx and DOSE scores, we plotted the receiver operating characteristic (ROC) curves and calculated their associated area under the curves (AUC) for the survival threshold of interest, i.e. 1 year. As a sensitivity analysis, the scores were compared on the first cohort (training and test set) without lung cancer.

All statistical analyses were carried out using STATA (version 15) and R (version 3.5.0).

## Results

### Characteristics of the COPD populations

There were 54,990 eligible COPD patients in the first cohort, from which the training and test datasets were drawn, of whom 21% died during study follow-up; median follow-up was 2.7 years (Additional file 1). The cohort had a median age of 70 years, around half were male, median BMI corresponding to overweight and a median FEV_1_ of 1.48 L (Table 1). All of the cohort had a history of at least one documented comorbidity. Only 1.2% of the cohort had a high COTE index. As might be expected, the cohort that died were slightly older, had a lower FEV_1_, had experienced more moderate and severe exacerbations, were on more inhaled medication and had in general more comorbidities. There were 4931 eligible COPD patients in the external validation dataset (Additional file 2: Figure S1), drawn from the second COPD cohort of whom 29% died during study follow-up; median follow-up was 2.1 years. The dataset had a median age of 71 years, 55% were males, and a median FEV_1_ was 1.52 L (Table 2). The patients that died were older, had a lower FEV_1_ and had more exacerbations and comorbidities.

**Table 1:** Demographic and clinical characteristics of the first COPD cohort (training and test datasets)

Characteristic	Training set		Test set		Died		Not died	
	*N*	%	*N*	%	*N*	%	*N*	%
Total	27,472	50.0	27,518	50.0	11,775	21.4	43,215	78.6
Mean age, years (SD)	69.9 (10.7)		70.0 (10.6)		76.6 (9.2)		68.1 (10.3)	
Gender (males)	14,869	54.1	12,603	45.9	4933	41.9	20,353	47.1
IMD quartiles								
1 (least deprived)	5251	19.2	5254	19.1	2237	19.0	8268	19.1
2	6926	25.3	6910	25.2	3021	25.7	10,815	25.0
3	7314	26.7	7341	26.8	3176	27.0	11,479	26.6
4	7891	28.8	7937	28.9	3327	28.3	12,501	28.9
Mean BMI, kg/m^2^ (SD)	27.5 (6.2)		27.5 (6.2)		26.0 (6.3)		28.0 (6.1)	
Mean FEV_1_, L (SD)	1.59 (0.7)		1.59 (0.7)		1.27 (0.60)		1.67 (0.69)	
GOLD stage								
1	10,117	43.1	10,231	43.5	3205	27.2	17,143	39.7
2	7850	33.5	7883	33.5	2776	23.6	12,957	30.0
3	4459	19.0	4407	18.8	2545	21.6	6321	14.6
4	1031	4.4	987	4.2	886	7.5	1132	2.6
MRC score								
1	14,553	53.5	14,524	53.5	3793	32.2	25,284	58.5
2	7011	25.8	7025	25.9	3262	27.7	10,774	24.9
3	4497	16.5	4482	16.5	3185	27.0	5794	13.4
4	1144	4.2	1118	4.1	1349	11.5	913	2.1
Smoking status								
Current	16,006	58.2	15,829	57.6	7272	61.8	24,563	56.8
Ex-smoker	11,512	41.8	11,643	42.4	4503	38.2	18,652	43.2
GP treated exacerbations								
1–2	7885	28.7	7738	28.2	3483	29.6	12,140	28.1
> 2	2230	8.1	2272	8.3	1291	11.0	3211	7.4
Hospitalised exacerbations								
1–2	1548	5.6	1609	5.9	1399	11.9	1758	4.1
> 2	149	0.5	118	0.4	181	1.5	86	0.2
Medications								
ICS	19,252	70.0	18,932	68.9	8937	75.9	29,247	67.7
LABA	17,638	64.1	17,456	63.5	8330	70.7	26,764	61.9
LAMA	13,522	49.1	13,585	49.5	6636	56.4	20,471	47.4
Influenza vaccination	22,663	82.4	22,649	82.4	9984	84.8	35,328	81.7
Pneumococcal vaccination	2195	27.5	1983	25.5	1059	9.0	3119	7.2
Comorbidities								
Myocardial infarction	2310	8.4	2162	7.8	1501	12.8	2971	6.7
Stroke	1393	5.1	1387	5.1	947	8.0	1833	15.6
Asthma	13,442	48.9	13,198	48.0	5937	50.4	20,703	175.8
Hypertension	13,263	48.3	13,130	47.7	6550	55.6	19,843	168.5
Atrial fibrillation (COTE)	2157	7.9	2222	8.1	1176	10.0	2663	22.6
Chronic kidney disease	2403	8.7	2391	8.7	2805	23.8	1989	16.9
Dementia	428	1.6	418	1.5	424	3.6	422	3.6
Anxiety (COTE)	6689	24.3	6715	24.4	2729	23.2	10,675	90.7
Depression	7941	28.9	8027	29.2	3004	25.5	12,964	110.1
Lung cancer (COTE)	1109	4.0	1055	3.8	1282	10.9	882	7.5
Cirrhosis (COTE)	119	0.4	118	0.4	76	0.6	161	1.4
Oesophageal cancer (COTE)	41	0.1	35	0.1	41	0.3	35	0.3
Diabetes	4599	16.7	4742	17.2	2408	20.5	6933	58.9
Pulmonary embolism	642	2.3	680	2.5	374	3.2	948	8.1
Heart failure (COTE)	1825	6.6	1775	6.5	1566	13.3	2034	17.3
Osteoporosis	2421	8.8	2392	8.7	11,480	97.5	3333	28.3
Coronary artery disease (COTE)	5620	20.5	5469	19.9	3361	28.5	7728	65.6
Pancreatic cancer (COTE)	5	0.0	5	0.0	< 5	< 0.1	6	0.1
Pulmonary fibrosis (COTE)	183	0.7	177	0.6	144	1.2	216	1.8
Breast cancer (COTE)	425	1.5	443	1.6	213	1.8	655	5.6
Gastric/duodenal ulcers (COTE)	230	0.8	229	0.8	121	1.0	338	2.9
Diabetes with neuropathy (COTE)	3894	14.2	4021	14.6	2073	17.6	5842	49.6
Blood tests								
CRP, mg/L mean (SD)	13.5 (29.5)		13.2 (29.6)		19.4 (36.8)		11.5 (24.5)	
Low albumin, g/L	1185	4.3	1176	4.3	1039	8.8	1322	11.2
Hb, g/dL mean (SD)	138.5 (41.4)		138.3 (37.5)		133.7 (40.0)		139.8 (39.2)	
Low platelets, × 10^9^/L	655	3.6	690	3.8	431	3.7	914	7.8
High platelets, × 10^9^/L	1374	7.5	1368	7.5	783	6.6	1959	16.6
Creatinine, μmol/L mean (SD)	88.9 (37.7)		88.2 (34.1)		98.1 (48.5)		85.7 (30.6)	
Bereavement	1337	4.9	1354	4.9	611	5.2	2080	17.7

*Hb* haemoglobin. Comorbidities included in COTE are indicated

**Table 2:** Demographic and clinical characteristics of the external dataset

Characteristic	External set		Died		Not died	
	*N*	%	*N*	%	*N*	%
Total	4931	100	1424	28.9	3507	71.1
Mean age, years (SD)	71.1 (11.0)		75.0 (9.0)		68.3 (11.1)	
Gender (males)	2718	55.1	912	64	1806	51.5
Mean BMI, kg/m^2^ (SD)	26.9 (6.5)		25.2 (6.2)		27.7 (6.4)	
Mean FEV_1_, L (SD)	1.52 (0.70)		1.34 (0.61)		1.73 (0.70)	
MRC score						
1	2433	49.3	478	33.6	1955	55.8
2	1224	24.8	373	26.2	851	24.3
3	926	18.8	387	27.2	539	15.4
4	348	7.1	186	13.1	162	4.6
Smoking status						
Ex-smoker	2657	53.9	793	55.7	1864	53.1
Current	2274	46.1	631	44.3	1643	46.9
Hospitalised exacerbations						
1–2	224	4.5	102	1.2	122	3.5
> 2	9	0.2	7	0.5	< 5	.
Comorbidities						
Stroke	368	7.5	144	10.1	224	6.4
Asthma	2452	49.7	736	51.7	1716	48.9
Hypertension						
Atrial fibrillation (COTE)	731	14.8	330	23.2	401	11.4
Chronic kidney disease	733	14.9	340	23.9	393	11.2
Dementia	210	4.3	76	5.3	134	3.8
Lung cancer (COTE)	80	1.6	50	3.5	30	0.9
Pulmonary fibrosis (COTE)	80	1.6	33	2.3	47	1.3
Blood tests						
Low albumin, g/L	369	7.5	153	10.7	216	6.2
Hb, g/dL mean (SD)	141.1 (67.7)		143.6 (102.0)		140.0 (47.1)	
Low platelets, × 10^9^/L	211	4.3	142	4.1	69	4.9
High platelets, × 10^9^/L	398	8.1	138	9.7	260	7.4
Creatinine, μmol/L mean (SD)	94.1 (45.4)		105 (52.5)		89.4 (41.3)	

### Prevalence of prognostic predictors

In the first primary care COPD cohort, there was < 5% missing data for the most commonly applied prognostic predictors, MRC dyspnoea score, BMI, smoking status, exacerbation history and age, except for FEV_1_ where there was 20% missing. Other predictors that had missing values were blood tests; CRP had 79% missing, albumin, haemoglobin, and platelets had around 30% missing and creatinine only had 23% missing. Only 16% of patients did not have a blood test within 12 months of their annual review, and only 3% were taken within 7 days either side of an exacerbation. There was 70% of patients with missing data for the pneumococcal vaccine. All other variables, unless derived from the abovementioned variables, were < 5% missing.

### Identification of the risk model

After imputation for the missing values and stepwise elimination, 18 different variables remained in the model, including age, BMI, FEV_1_, severe exacerbations, smoking status, multiple comorbidities, haemoglobin and platelets (Table 3).

**Table 3:** Estimated beta coefficients and their standard errors (SE) for the final Cox proportional hazards model

	Coefficient	SE	*p* value
Continuous variables			
Age	0.040	0.004	< 0.0001
BMI	− 0.047	0.006	< 0.0001
Creatinine	0.001	0.001	< 0.05
Haemoglobin	− 0.006	0.001	< 0.0001
FEV1	− 0.433	0.078	< 0.0001
Categorical variables			
Asthma	− 0.155	0.057	< 0.01
Atrial fibrillation	0.450	0.077	< 0.0001
No CKD	− 0.314	0.143	< 0.05
Current smoker	0.179	0.061	< 0.05
Dementia	0.391	0.137	< 0.01
Female	− 0.331	0.069	< 0.0001
Hospitalisations			
1–2/year	0.517	0.081	< 0.0001
> 2/year	0.980	0.181	< 0.0001
Low albumin	0.531	0.089	< 0.0001
Lung cancer	1.063	0.135	< 0.0001
Lung fibrosis	0.794	0.219	< 0.0001
MRC			
2	0.577	0.130	< 0.0001
3	0.890	0.133	< 0.0001
4	1.599	0.142	< 0.0001
Platelets			
Normal	− 0.394	0.131	< 0.01
High	− 0.208	0.161	> 0.05
Stroke	0.235	0.097	< 0.05

The marginal predictions for the risk of death at 1 year were obtained by the following equation

$$ P\left(\mathrm{death}\ \mathrm{at}\ 1\ \mathrm{year}\right)=1-{(0.9837)}^{\exp \left(\mathrm{prognostic}\ \mathrm{index}\right)}, $$

where the baseline survival is estimated by means of a fractional polynomial and the prognostic index is the linear combination of the coefficients given in Table 3 with the values of the corresponding variables.

### Validation of the risk model

The predictive performance and calibration of the BARC index was high in the test dataset and satisfactory in the external dataset (test set: C-index 0.79, 95% CI 0.78–0.81; D-statistic 1.9, 95% CI 1.8–2.0; calibration slope 0.95, 95% CI 0.90–0.99, and external dataset: C-index 0.67, 95% CI 0.65–0.70; D-statistic 0.98, 95% CI 0.83–1.14; calibration slope 0.54, 95% CI 0.45–0.64) (Table 4). We depict the observed and fitted survival probabilities, with pointwise 95% confidence intervals for the latter, at 3, 6 and 9 months, other than at 1 year, to give a visual trend of the survival probabilities. The graphical analysis confirms the satisfactory calibration of the BARC index (Additional file 3: Figure S2), even if the predictions in some of the groups were slightly higher than the observed.

**Table 4:** Validation at 12 months using the test and external validation datasets

	Mean	SE	95% CI	Mean	SE	95% CI
	Test set			External validation		
Harrell’s C-index	0.794	0.006	0.782–0.807	0.671	0.012	0.647–0.695
D-statistic	1.865	0.048	1.770–1.959	0.983	0.080	0.826–1.141
Calibration slope	0.947	0.024	0.900–0.993	0.544	0.046	0.454–0.635

### Comparing mortality between that observed and predicted

**Fig. 1:**
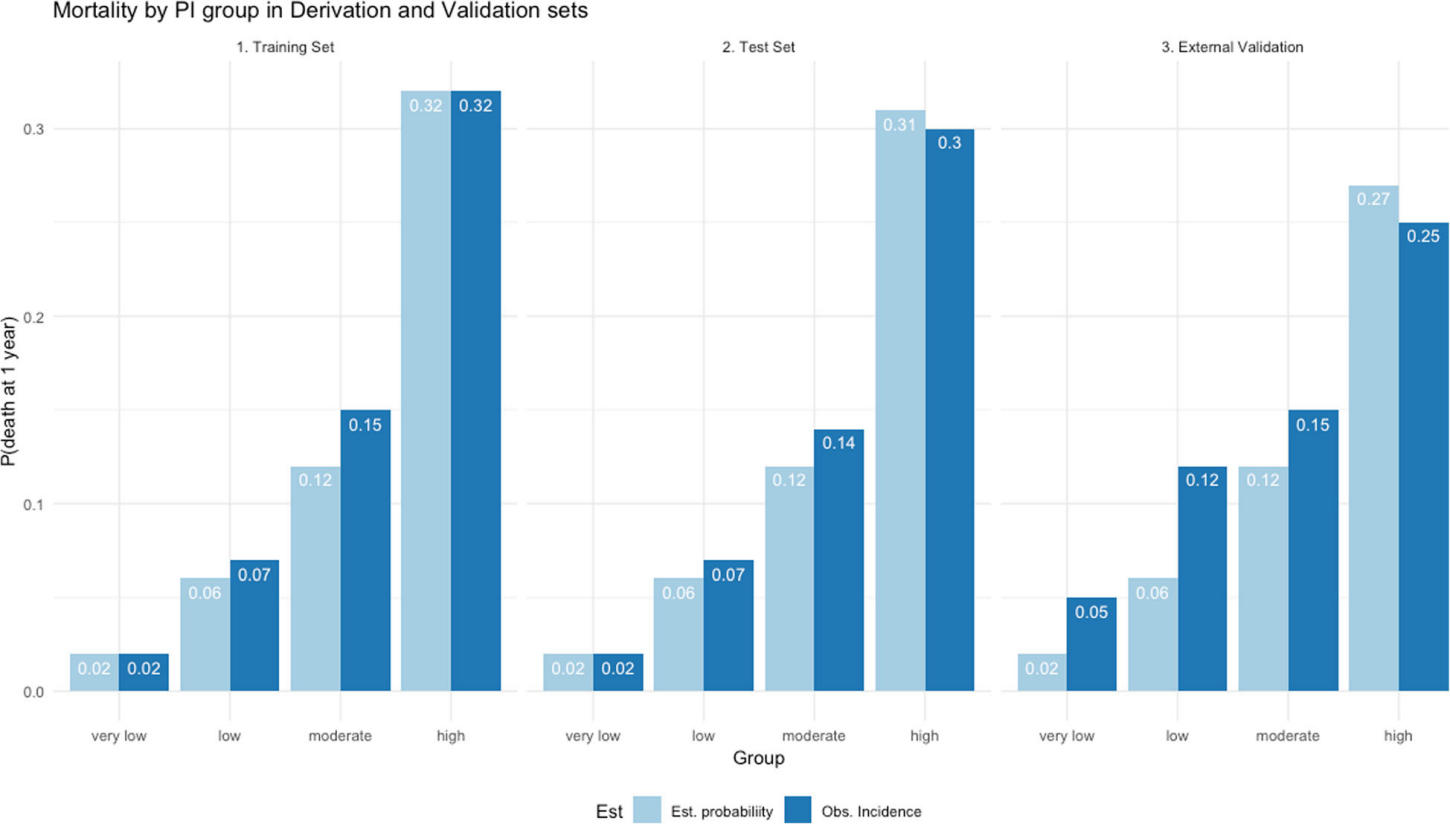
Mortality probability by PI group in training, test and external validation datasets

There was an increasing probability of death with each increasing risk group (Fig. 1). The BARC index estimated the probability of dying to within 1% of the observed probability in the high-risk group, in the training and test sets, and within 5% in the external dataset.

### Comparing the BARC to ADO, BODEx and DOSE

**Fig. 2:**
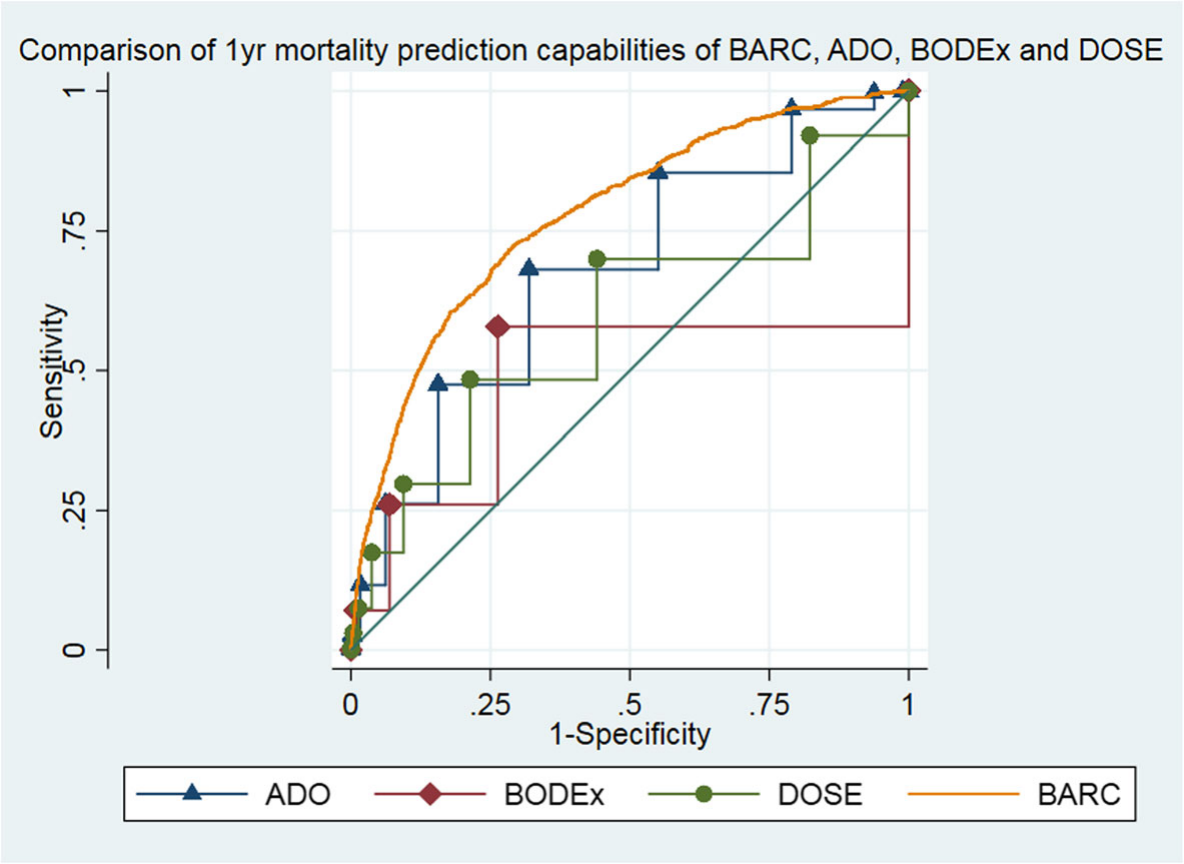
Receiver operating curves comparing the BARC index with ADO, BODEx and DOSE indexes

The ROC curve of the BARC index was consistently above any of the curves associated with both ADO, BODEx and DOSE scores, showing that our model performed better in the test dataset than the three scores (Fig. 2 and Additional file 4: Figure S3). This result is confirmed by the associated AUCs and their corresponding 95% confidence intervals (Table 5). BARC index still performed better in the sensitivity analysis, removing lung cancer patients (Additional file 1: Tables S2-S4).

**Table 5:** AUCs for the BARC, ADO, BODEx and DOSE indexes

	AUC	SE	95% CI	AUC	SE	95% CI
Prognostic index	First cohort			External validation		
ADO	0.675	0.010	0.655–0.694	0.568	0.014	0.541–0.595
BODEx	0.483	0.015	0.453–0.512	0.413	0.017	0.379–0.447
DOSE	0.591	0.012	0.568–0.614	0.515	0.015	0.485–0.546
BARC	0.781	0.009	0.764–0.792	0.695	0.012	0.671–0.719

## Discussion

From a large cohort of primary care COPD patients, we have derived a 12-month mortality predictive model, the BARC index, with acceptable discrimination and calibration when externally validated. The predictive performance of the model also compared favourably to the commonly used ADO, DOSE and BODEx indexes. The BARC index is comprised of variables commonly included in established predictive indexes, such as airway obstruction, age, smoking status and dyspnoea assessment, as well as several comorbidities and blood biomarkers linked to general health (including serum albumin and haemoglobin).

A significant difference between our more favourable model and established scores is the addition of comorbidities. The presence of comorbid disease is common, with at least 80% of COPD patients estimated to have one or more additional chronic disorders; indeed, those within 1 year of death have an even larger proportion with comorbid disease [7, 29]. It is also associated with significantly increased mortality; up to two thirds of deaths are thought to be from comorbid disease not COPD [12, 15, 30]. Perhaps unexpectedly, most cardiovascular comorbidities were not included in the model at the 5% significance level; however, this may be because this model addressed shorter-term 12-month mortality whereas cardiovascular disease has relatively longer-term effects, than some other comorbidities, such as cirrhosis, lung cancer and cerebrovascular disease that were included. Furthermore, cardiovascular mortality continues to decrease [31]. The specific comorbidities index (COTE) uses 12 comorbidities, but no respiratory parameters, and provides a good 5-year mortality prediction [15]. However, COTE has not been assessed for predicting mortality at 1 year, and as it was derived in secondary care, it requires specialised knowledge on disease status that is not always available. In this respect, the CODEX index (based on the Charlson index and BODEx), derived from a selective cohort of hospitalised COPD patients, also requires in-depth knowledge on comorbidities [18]. In comparison, many variables that are associated with COPD severity, including medication use, moderate exacerbations and GOLD staging, were not included in the model at the 5% significance level. This information in itself points to the complexity of understanding COPD mortality and highlights again the influence of comorbid conditions on mortality.

One advantage of the BARC index is that it is practical, and user-friendly, as it incorporates routinely collected data easily available within primary care, which could also allow the risk score to be embedded in the electronic healthcare records system. In addition, because it was derived and validated in two large nationally representative COPD populations, and nearly 90% of UK population is registered in primary care, this aids the generalisability of the risk score to all COPD populations. The cohorts used had similar mortality rates to other COPD cohorts (data not shown) [11, 32]. However, the generalisability could have been reduced as we used an annual review as the arbitrary time point from which to start the study; 20% of the cohort did not have one during their study period, and this was largely due to their short length of research quality data available (i.e. only had just over 1 year of CPRD data therefore not long enough to have 1 year of data and an annual review) rather than lack of attendance to their annual review. This generalisability issue was overcome as the external dataset contained patients without an annual review during that time period. Another possible limitation of the derivation of the risk score is that five variables (FEV1, albumin, haemoglobin, platelets and creatinine) had to be imputed due to missing data, which potentially could have led to misclassification, though the percentage missing was only around 10 to 30%. The low percentage of missing data in the first cohort was likely due to some selection bias as these patients all had an annual review; there was higher percentage missing in the second cohort, with 15% missing FEV_1_ and 50% missing MRC dyspnoea score. In the first cohort, many of the missing variables appeared to be missing due to a relatively short follow-up period before death (in the UK FEV_1_ is routinely measured every 18 months); nevertheless, FEV_1_ can readily be measured by spirometry if required for the index. Although blood tests were missing from some patients, these provided significant predictive value to the model and were mostly performed less than a year before the annual review date. Moreover, we feel it is likely in patients where a GP is considering this index, they will have had a blood test in the recent past; if not, this information can easily be obtained from a simple single blood test. A strength of this study is the use of such a large cohort of patients to derive the model from; this also provided the power to assess less-common comorbidities (including cirrhosis and dementia) as statistically significant prognostic markers that may not have been found in a smaller sample size.

Information on the end of life has been identified as of intrinsic interest to patients, carers and healthcare professionals, but the lack of the ability to approximately predict mortality is thought to be one of the key barriers to providing this information. Therefore, the identification of this accurate, user-friendly, predictive model that is applicable in primary care, could aid communication, shared decision-making and ultimately a palliative care approach directed from primary care. Our findings suggest the currently used predictive scores may be too simple and that incorporating more clinical variables, in particular comorbidities, significantly improves predictive performance. Of course, a risk score only aids decision-making, and physicians should use their clinical acumen and discuss with patients and their families to decide when palliative care is appropriate (it may be appropriate long before the last year of life); a risk score should not be used in isolation as a screening tool for palliative care [28].

## Conclusions

This is the first published prognostic tool designed to predict all-cause mortality in patients with COPD within 12 months of death. In addition, its applicability in primary care, and validation in a large general COPD cohort, gives the BARC index significant clinical and practical advantages over previously identified risk indices.

## Additional files

Additional file 1: **Tables S1-S4.** **Table S1.** Time scale of when variables data collected according to index date (annual review for training and test dataset and 12 months after eligibility date for external validation dataset). **Table S2.** AUCs for the BARC, ADO, BODEx and DOSE indexes in the sensitivity analysis, removing patients with lung cancer from the test dataset. **Table S3.** Model performance in the sensitivity analysis, removing patients with lung cancer from the external dataset. **Table S4.** AUCs for the BARC, ADO, BODEx and DOSE indexes in the sensitivity analysis, removing patients with lung cancer from the external dataset. (DOCX 19 kb)

Additional file 2: **Figure S1.** Flow diagram of inclusion criteria and patient numbers. (PNG 38 kb)

Additional file 3: **Figure S2.** Calibration of a Cox model in the test datasets. Smooth dashed lines represent predicted survival probabilities, and vertical capped lines denote Kaplan–Meier estimates with 95% confidence intervals. Four prognosis groups are plotted (from darkest to palest): the “very low” risk group, the “low” risk group, the “moderate” risk group and the “high” risk group. (PNG 239 kb)

Additional file 4: **Figure S3.** Receiver operating curves comparing the BARC index with ADO, BODEx and DOSE indexes in the external dataset. (PNG 56 kb)

## Abbreviations

ADO: Age, dyspnoea and obstruction
BARC: Body mass index, blood test, age, respiratory variable and comorbidities
BODEx: Body mass index, obstructive, dyspnoea and exacerbations
COPD: Chronic obstructive pulmonary disease
CPRD: Clinical Practice Research Datalink
CRP: C-reactive protein
DOSE: Dyspnoea, obstruction, smoking and exacerbations
GP: General practitioner
Hb: Haemoglobin
HES: Hospital Episode Statistics
ICS: Inhaled corticosteroid
IMD: Index of Multiple Deprivation
LABA: Long-acting beta agonist
LAMA: Long-acting muscarinic antagonist
NHS: National Health Service
ONS: Office of National Statistics
SD: Standard deviation
UK: United Kingdom
