# Supplementary material for: Predicting COPD 1-year mortality using prognostic predictors routinely measured in primary care
Source: BMC Med. 2019 Apr 5;17:73. doi: 10.1186/s12916-019-1310-0 (PMC6449897; doi:10.1186/s12916-019-1310-0)
Supplement: Supplementary file 1 — Tables S1-S4. Table S1. Time scale of when variables data collected according to index date (annual review for training and test dataset and 12 months after eligibility date for external validation dataset). Table S2. AUCs for the BARC, ADO, BODEx and DOSE indexes in the sensitivity analysis, removing patients with lung cancer from the test dataset. Table S3. Model performance in the sensitivity analysis, removing patients with lung cancer from the external dataset. Table S4. AUCs for the BARC, ADO, BODEx and DOSE indexes in the sensitivity analysis, removing patients with lung cancer from the external dataset. (DOCX 19 kb) [file 12916_2019_1310_MOESM1_ESM.docx]

**Table S1** Time scale of when variables data collected according to index date (annual review for training and test dataset and 12 months after eligibility date for external validation dataset).

| **Demographic & Clinical Characteristics** | **Variable time scale** |
| --- | --- |
| Age at start date | At index date |
| Gender | At index date |
| IMD | At index date |
| BMI | Latest pre-ID |
| Smoking | Nearest to time of ID |
| MRC | Within 1.5 years ID |
| FEV1 | Latest pre-ID |
| GOLD | Latest pre-ID |
| Exacerbation history | 1 year pre-ID |
| Influenza vaccination | 1 year pre-ID |
| Pneumococcal vaccination | 5 year pre-ID |
| **Medications** |  |
| ICS ever prescription | 1 year pre-ID |
| LABA ever prescription | 1 year pre-ID |
| LAMA ever prescription | 1 year pre-ID |
| LTOT | Within 1.5 years AR |
| **Co-morbidities** |  |
| Myocardial infarction | Ever pre-ID |
| Stroke | Ever pre-ID |
| Heart failure | Ever pre-ID |
| Lung cancer | Ever pre-ID |
| Angina | Ever pre-ID |
| Asthma | Ever pre-ID |
| Osteoporosis | Ever pre-ID |
| Diabetes | Ever pre-ID |
| Depression | Ever pre-ID |
| Anxiety | Ever pre-ID |
| Hypertension | Ever pre-ID |
| Dementia | Ever pre-ID |
| Atrial fibrillation | Ever pre-ID |
| Cirrhosis | Ever pre-ID |
| Pulmonary embolism | Ever pre-ID |
| Gastric/duodenal ulcers | Ever pre-ID |
| Diabetes with neuropathy | Ever pre-ID |
| Breast cancer | Ever pre-ID |
| Oesophageal cancer | Ever pre-ID |
| Coronary artery disease | Ever pre-ID |
| Pancreatic cancer | Ever pre-ID |
| Pulmonary fibrosis | Ever pre-ID |
| **Blood test results** |  |
| CRP | Within 1.5 years ID |
| Albumin | Within 1.5 years ID |
| Haemoglobin | Within 1.5 years ID |
| Platelets | Within 1.5 years ID |
| Creatinine | Within 1.5 years ID |
| CKD (based on creatinine) | Within 1.5 years ID |
| **Other variables of interest** |  |
| Bereavement | Within 5 years ID |

**Table S2** AUCs for the BARC, ADO, BODEx and DOSE indexes in the sensitivity analysis, removing patients with lung cancer from the test dataset.

|  | **AUC** | **SE** | **95% CI** |
| --- | --- | --- | --- |
| **ADO** | 0.678 | 0.010 | 0.658-0.698 |
| **BODEx** | 0.480 | 0.015 | 0.450-0.511 |
| **DOSE** | 0.591 | 0.012 | 0.567-0.616 |
| **BARC** | 0.778 | 0.009 | 0.761-0.796 |

**Table S3** Model performance in the sensitivity analysis, removing patients with lung cancer from the external dataset.

|  | **mean** | **se** | **lower 95% CI** | **upper 95% CI** |
| --- | --- | --- | --- | --- |
| **Harrell's C index** | 0.662 | 0.013 | 0.636 | 0.688 |
| **D-statistic** | 0.913 | 0.084 | 0.747 | 1.078 |
| **Calibration Slope** | 0.503 | 0.047 | 0.410 | 0.596 |

**Table S4** AUCs for the BARC, ADO, BODEx and DOSE indexes in the sensitivity analysis, removing patients with lung cancer from the external dataset.

|  | **AUC** | **SE** | **95% CI** |
| --- | --- | --- | --- |
| **ADO** | 0.578 | 0.015 | 0.548-0.607 |
| **BODEx** | 0.429 | 0.018 | 0.393-0.464 |
| **DOSE** | 0.521 | 0.016 | 0.489-0.553 |
| **BARC** | 0.676 | 0.014 | 0.648-0.704 |
