# Supplementary figures and images for: Predicting COPD 1-year mortality using prognostic predictors routinely measured in primary care
Source: BMC Med. 2019 Apr 5;17:73. doi: 10.1186/s12916-019-1310-0 (PMC6449897; doi:10.1186/s12916-019-1310-0)

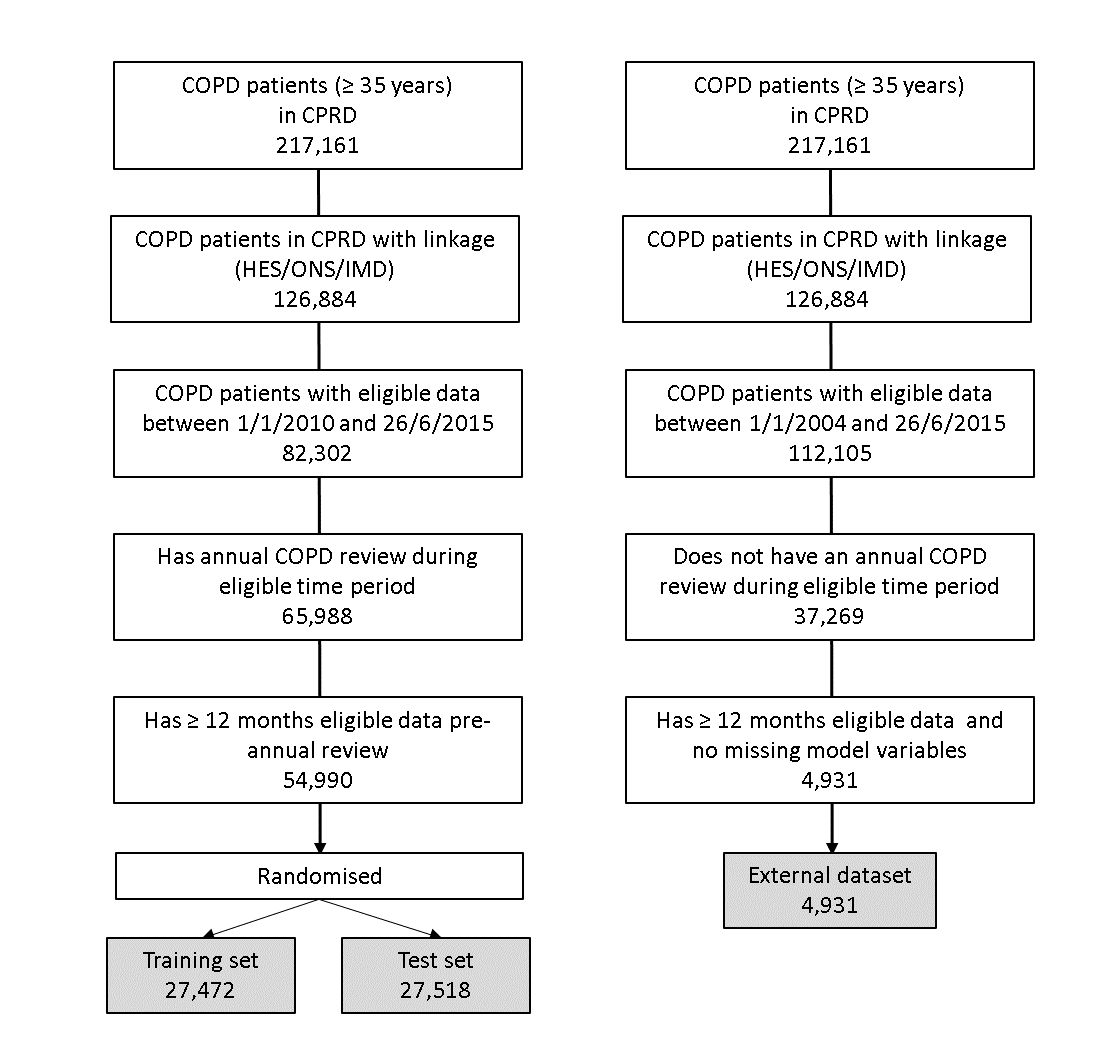

Supplement: Supplementary file 2 — Figure S1. Flow diagram of inclusion criteria and patient numbers. (PNG 38 kb) [file 12916_2019_1310_MOESM2_ESM.png]

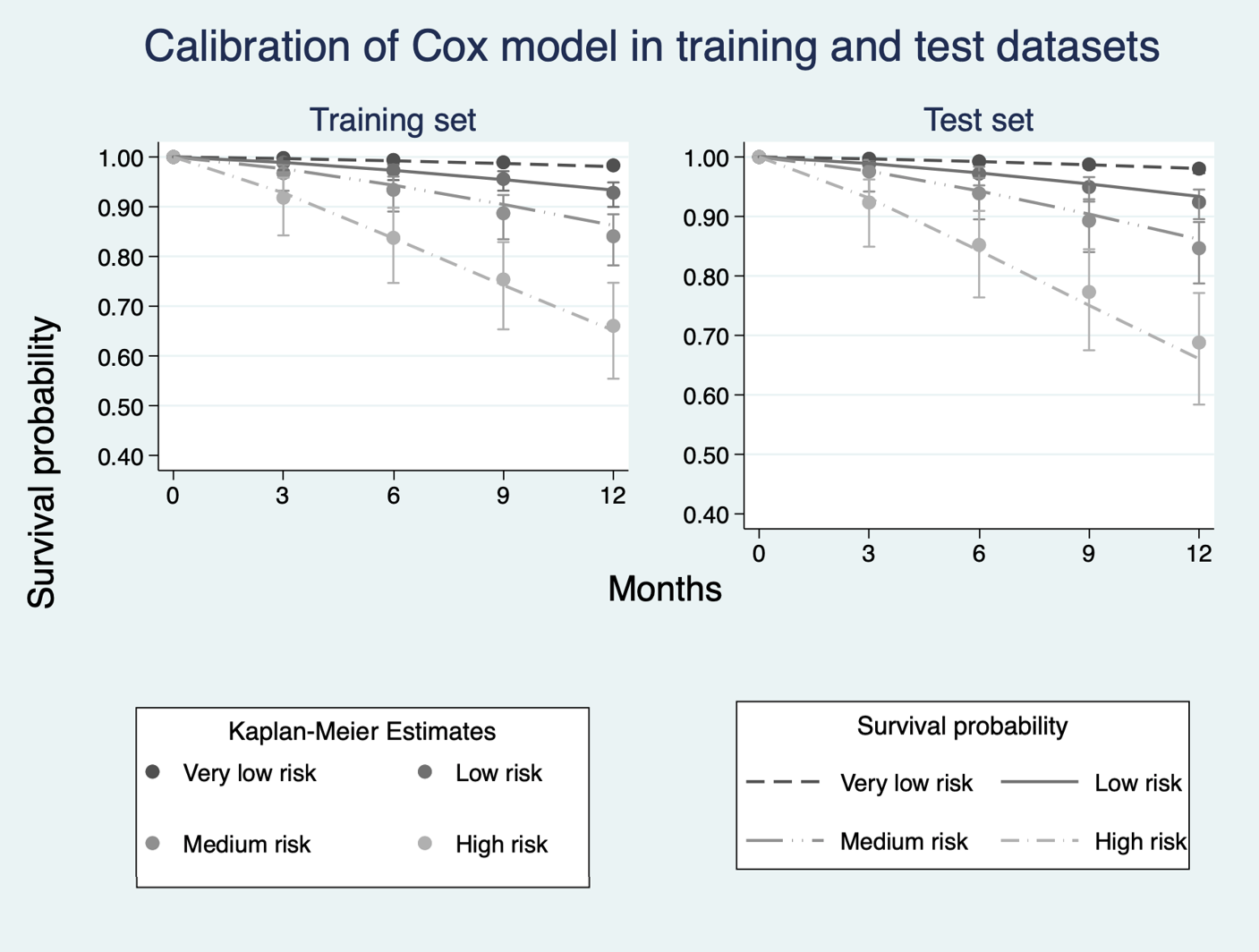

Supplement: Supplementary file 3 — Figure S2. Calibration of a Cox model in the test datasets. Smooth dashed lines represent predicted survival probabilities, and vertical capped lines denote Kaplan–Meier estimates with 95% confidence intervals. Four prognosis groups are plotted (from darkest to palest): the “very low” risk group, the “low” risk group, the “moderate” risk group and the “high” risk group. (PNG 239 kb) [file 12916_2019_1310_MOESM3_ESM.png]

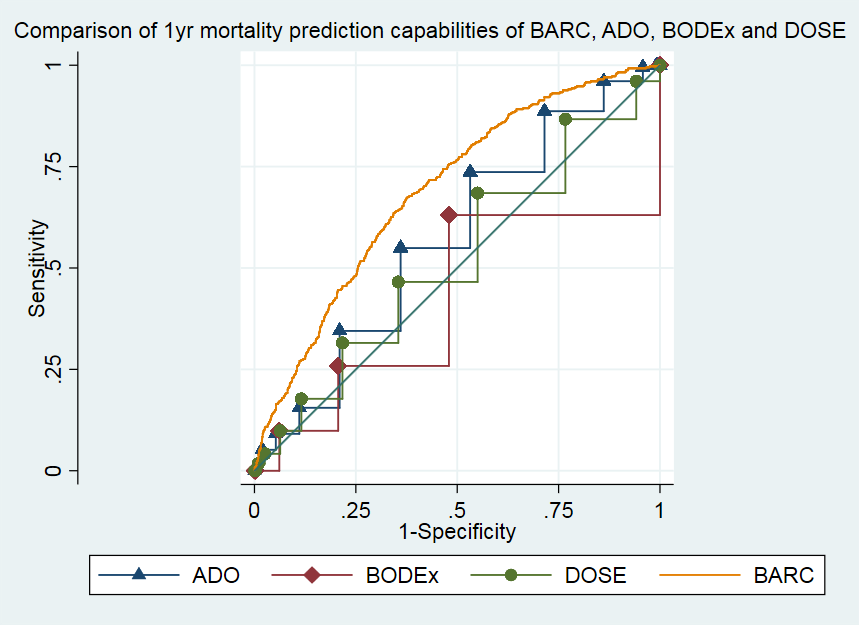

Supplement: Supplementary file 4 — Figure S3. Receiver operating curves comparing the BARC index with ADO, BODEx and DOSE indexes in the external dataset. (PNG 56 kb) [file 12916_2019_1310_MOESM4_ESM.png]
